# MnO Nanoparticle@Mesoporous Carbon Composites Grown on Conducting Substrates Featuring High-performance Lithium-ion Battery, Supercapacitor and Sensor

**DOI:** 10.1038/srep02693

**Published:** 2013-09-18

**Authors:** Tianyu Wang, Zheng Peng, Yuhang Wang, Jing Tang, Gengfeng Zheng

**Affiliations:** 1 Laboratory of Advanced Materials, Department of Chemistry, Fudan University, Shanghai, China

## Abstract

We demonstrate a facile, two-step coating/calcination approach to grow a uniform MnO nanoparticle@mesoporous carbon (MnO@C) composite on conducting substrates, by direct coating of the Mn-oleate precursor solution without any conducting/binding reagents, and subsequent thermal calcination. The monodispersed, sub-10 nm MnO nanoparticles offer high theoretical energy storage capacities and catalytic properties, and the mesoporous carbon coating allows for enhanced electrolyte transport and charge transfer towards/from MnO surface. In addition, the direct growth and attachment of the MnO@C nanocomposite in the supporting conductive substrates provide much reduced contact resistances and efficient charge transfer. These excellent features allow the use of MnO@C nanocomposites as lithium-ion battery and supercapacitor electrodes for energy storage, with high reversible capacity at large current densities, as well as excellent cycling and mechanical stabilities. Moreover, this MnO@C nanocomposite has also demonstrated a high sensitivity for H_2_O_2_ detection, and also exhibited attractive potential for the tumor cell analysis.

Manganese oxides (MnO_x_) are a class of transition metal oxides, including MnO, MnO_2_, Mn_3_O_4_, Mn_2_O_3_, which are endowed with rich oxidation states and chemistry^1^,^2^,^3^. The electron transfer of MnO_x_ structures, along with the fast development of versatile structures controlled during the growth, has offered substantial potentials in many application fields, including catalysis^4^, chemical/biological sensing^5^,^6^, and energy storage^7^,^8^. Manganese oxides are promising candidates for active electrode materials, due to their high specific capacitance, low cost, abundance and environmentally benign nature^1^. For instance, MnO_2_ and MnO have high theoretical capacities of ~1232 and 755 mAh g^−1^ as lithium-ion battery (LIB) anodes, respectively^9^. For supercapacitors, the nanostructured manganese oxides have presented great capacitance retention upon cycling^10^. Nonetheless, these performances are still limited due to, in general, the low electrical conductivity, low rate capability, and suboptimal structural stability of MnO_x_^2^,^11^. A variety of approaches, such as nanostructure fabrication^10^,^11^,^12^,^13^,^14^, chemical modification^15^,^16^, and incorporation with high surface-area, conductive materials^17^,^18^, have been explored to improve the performance of MnO_x_-based electrodes. For instance, Wang *et al*. reported a solution approach of growing Mn_3_O_4_ nanoparticles on reduced graphene oxide sheets, with a high specific capacity up to ~900 mAh g^−1^ as LIB anodes^19^. Mallouk and coworkers developed a template-free hydrothermal synthesis of graphene/Mn_3_O_4_ nanorod composites from KMnO_4_ and ethylene glycol, which showed an enhanced capacitance and long cycle stability over free Mn_3_O_4_ nanorods^20^. Jiang *et al*. reported a sol-gel method for the growth of MnO_x_ nanoparticle/mesoporous carbon/MnO_x_ hybrid nanowires, where a high specific capacitance of 266 F g^−1^ at 1 A g^−1^ was obtained^21^. In addition to the solution methods, the chemical vapor deposition (CVD) method has also been used to deposit carbon coating on the surface of porous MnO microspheres, which are obtained by decomposing Mn precursors such as MnCO_3_^22^. For biosensing, one well-known example is that MnO_2_ is good catalyst for decomposition of H_2_O_2_, which is an important intermediate or product of many biochemical reactions and has a well-established relationship with numerous biological processes^23^. The detection of H_2_O_2_ has been demonstrated previously with MnO_2_ nanoparticles^24^, which waives the need of electrode modification with enzymes. Recently, a sensitive detection of H_2_O_2_ was reported by a MnO_2_/graphene oxide nanocomposite, with a low detection limit of 0.8 μM^25^.

In spite of these research progresses, several main challenges still need to be addressed for optimizing the conductance and rate performance of MnO_x_-based electrodes. For the direct growth of conducting carbon nanostructures including carbon nanotubes and graphene by CVD, the requirement of high reaction temperatures precludes the use of a majority of substrates, especially flexible substrates. In addition, the loading density of MnO_x_@C composites with MnO_x_ directly grown on conducting substrates is usually restricted, due to the ultrathin film of the MnO_x_ backbones^21^. On the other hand, when the pre-formed MnO_x_@C nanostructures is coated on a current collector, it often needs adding auxiliary binders and/or conductive reagents, such as conducting polymer^11^ and acetylene black^21^, which takes additional fabrication steps and cost, as well as reduces the effective mass percentage of electroactive materials. Furthermore, the lack of nanoscale pores that can allow for efficient mass transport towards and from the active MnO_x_ sites also limits their rate capability for energy storage, as well as sensitive molecular detection. Recently, an *in situ* method based on a free-radical polymerization in the presence of metal oxide precursors was reported to produce a cross-linked polymer network incorporated with Fe_3_O_4_ or MnO nanoparticles (NPs), which can be thermally converted into uniform NP@C nanocomposites for LIB application^26^. However, this approach is still constrained by the use of conducting polymer as a binder and structural directing agent, and the obtained reversible capacity of the MnO@C nanocomposite is still far from the theoretical values.

In this paper, we demonstrate a facile method for direct coating of MnO organic precursor solution onto substrates without conducting polymer binders, followed by thermal treatment to grow monodispersed, ~10 nm-diameter MnO NPs embedded in a mesoporous carbon matrix (Figure 1). This MnO@C nanocomposite allows for efficient charge transfer, in which the carbon matrix serves as the major pathways for enhanced charge transport from MnO NPs. In addition, the mesopores of the carbon matrix offer efficient mass transport for electrolyte solution and chemical species, while at the same time providing volume buffer for MnO NPs during lithiation/delithiation, thus leading to excellent rate capabilities and cycling stability of this MnO@C nanocomposite. Furthermore, this approach can be applied to a large variety of substrates, including flexible ones for potential applications in portable energy storage and sensing devices. As proofs-of-concept, LIB anodes made of this monodispersed MnO@C nanocomposite display excellent reversible capacities of over 800 and 520 mAh g^−1^ at current densities of 0.1 and 2 A g^−1^, respectively. Supercapacitors made of this MnO@C nanocomposite exhibit stable capacitances of 160 and 40 F g^−1^, at current densities of 1 and 40 A g^−1^, respectively, which also show excellent mechanical stability over repeated folding and stretching. Finally, this MnO@C nanocomposite demonstrates sensitive electrical response to H_2_O_2_ in buffer solutions, and has been applied to interrogate the H_2_O_2_ concentration in cellular assays for tumor cell analysis.

## Results

### Synthesis and structural characterization

**Figure 2:**
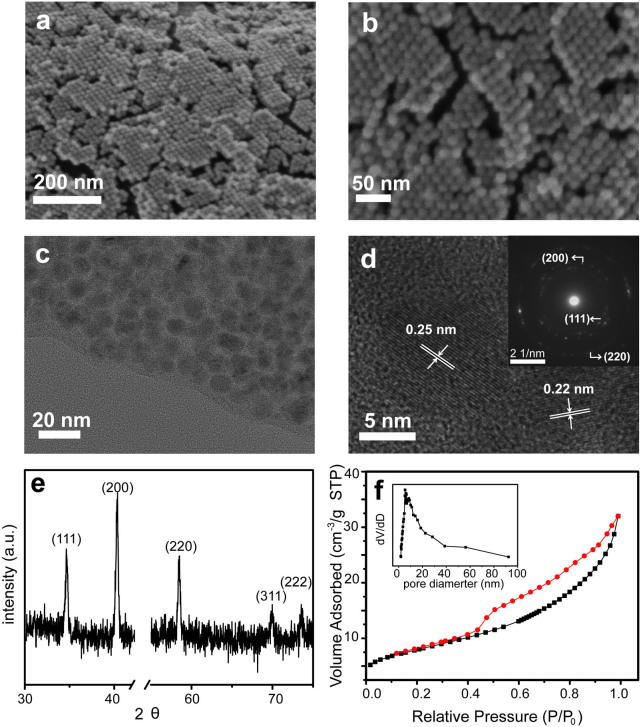
(a, b) SEM images of MnO@C nanocomposite on a Ni foam substrate. (c) TEM, (d) HRTEM images and (inset) SAED pattern of MnO@C nanocomposites. (e) XRD pattern of MnO@C nanocomposite on a Ni foam substrate. (f) N_2_ sorption isotherm and corresponding pore size distribution curve (inset) of MnO@C nanocomposite.

The MnO@C nanocomposite is synthesized by a modified method^27^, in which a Mn-oleate precursor solution is directly coated on a conducting substrate, followed by thermal calcination at 550°C in Ar (Methods). After the solution coating, the substrate surface is covered by a uniform layer of brown color waxy solid, which turns black after thermal treatment. This process can be applied to a large variety of substrates, including Ni foam, Ti foil, carbon fiber, silicon wafer, fluorine-doped tin oxide (FTO) glass, and so on (Figure S1), indicating the general availability of this two-step coating-conversion method. The film thickness resulted from a single coating is ~300 nm (Figure S2a), corresponding to a net mass per area of ~1.24 mg cm^−2^ (Figure S2b). Repeated coating of the Mn-oleate precursor solution on the substrate leads to an almost linear increase of the film mass with the coating times. High-resolution scanning electron microscopy (SEM) images show that the substrate is covered with a film of multi-layered, hexagonally closed-packed spherical NP arrays, with a uniform size distribution of ~10 nm (Figure 2a, b). Random, short cracks are observed over the film surface between different NP domains, which may result from the volume contraction of the nanocomposite during thermal treatment. Nonetheless, most of the NPs are still closed packed and the majority of the film is continuous. Transmission electron microscopy (TEM) images exhibit that these monodispersed NPs are embedded in an amorphous carbon matrix, in which each NP is coated by the carbon layer without being aggregated with adjacent ones (Figure 2c). The average diameter of the NPs is 10 ± 2 nm. High-resolution TEM (HRTEM) images reveal that each NP is single crystalline with few observable structure defects (Figure 2d). Well-resolved lattice fringes are observed from these nearly spherical NPs, which correspond to *d*-spacing values of 0.25 and 0.22 nm, consistent with the (111) and (200) planes reported for single crystal MnO^26^. The select area electron diffraction (SAED) pattern shows a poly-crystalline diffraction pattern, due to the different orientation from various MnO NPs (Figure 2d, inset). The first three diffraction rings of the SAED pattern correspond to the (111), (200) and (220) lattice planes.

The structure and phase purity of this MnO@C nanocomposite grown on Ni foam is further characterized by X-ray diffraction (XRD), which displays well-resolved diffraction peaks at 34.9°, 40.5°, 58.7°, 70.2°, 73.8° (Figure 2e). These peaks are well indexed as the 111, 200, 220, 311, and 222 reflections of MnO (JCPDS Card no. 07-0230), in good accord with the HRTEM and SAED results. No additional peaks other than the Ni foam are observed (Figure S3), indicating the high purity of the obtained MnO NPs. The carbon coating is confirmed by the Raman spectra with two bands at 1578 and 1364 cm^−1^ (Figure S4), attributed to the G-band and D-band of carbon, indicating the existence of both sp^2^ and sp^3^ carbons, respectively. The amount of MnO in the nanocomposite is quantified as ~84.3%, measured by inductively coupled plasma (ICP). Furthermore, the N_2_ sorption isotherm of the MnO@C nanocomposite shows a typical type-IV curve and a distinct condensation step (Figure 2f), indicating the existence of mesopore structures^28^,^29^. The surface area (*S*_BET_) is calculated as 28 m^2^ g^−1^, which is comparable to MnO_2_ nanostructures produced under similar temperatures^1^. The pore size derived from the adsorption branch shows a relatively narrow distribution of 5–40 nm (Figure 2f, inset). The surface area and large pore size are beneficial for providing sufficient interface between the electroactive materials and the electrolyte.

### Lithium-ion battery anode

**Figure 3:**
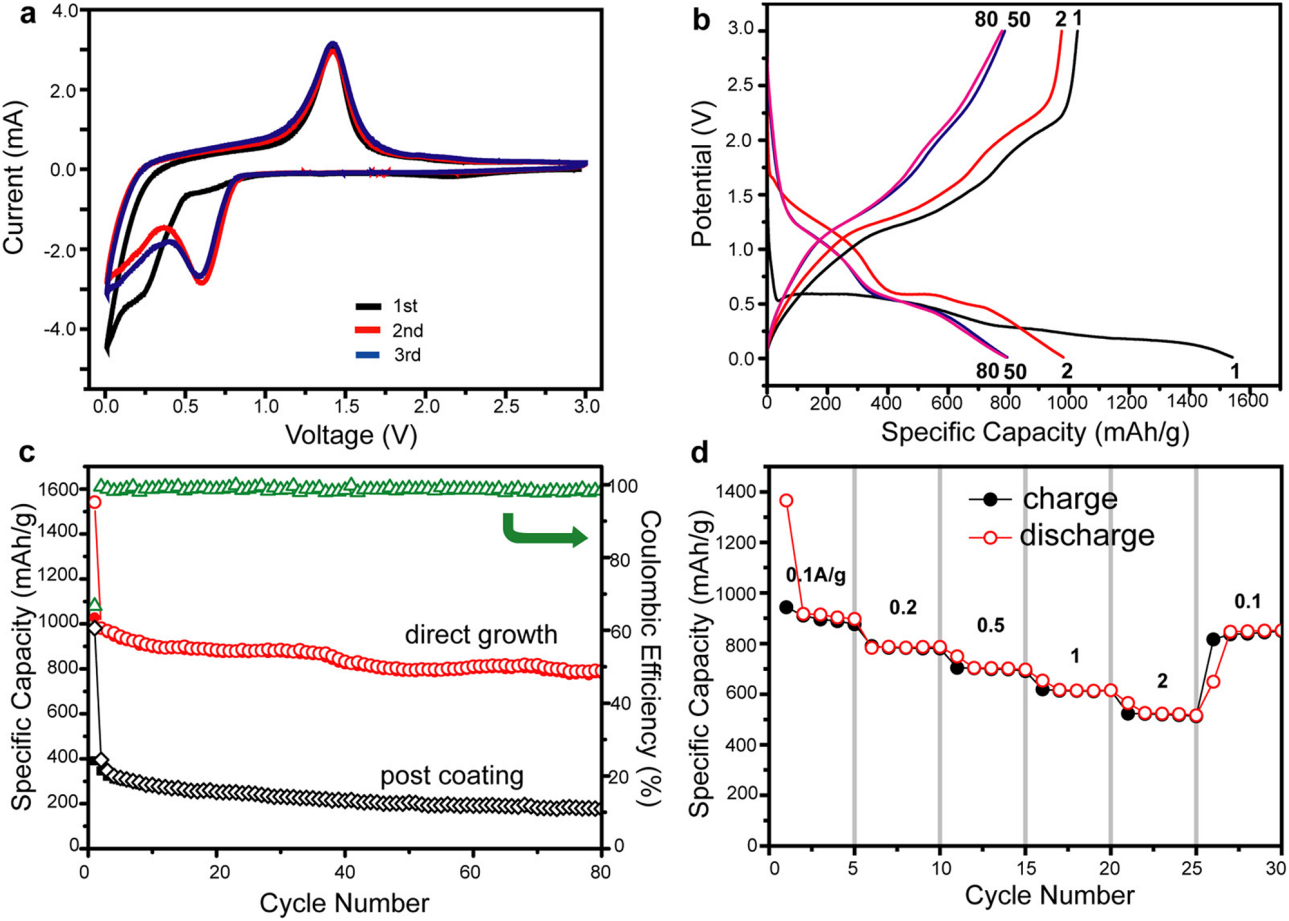
LIB measurement of MnO@C nanocomposite on Ni foam. (a) Cyclic voltammograms for the first 3 cycles. (b) Voltage profiles at a current density of 100 mA g^−1^ for the 1^st^, 2^nd^, 50^th^ and 80^th^ discharge/charge cycles. (c) Cycling performances of directly growth (red curve) and post-coating (black curve) of MnO@C nanocomposite on Ni foams. The Coulombic efficiency of the direct growth method is also displayed (green curve). (d) Capacity retention at different charge/discharge rates from 0.1–2 A g^−1^.

The electrochemical storage capacity of the obtained MnO@C nanocomposites is first investigated for LIB anodes, where the precursor solution is directly coated on a Ni foam substrate before thermal treatment, without adding any conducting polymers or binders. Cyclic voltammetry (CV) tests are first carried out to characterize the electrochemical reaction (Figure 3a). In the reduction half cycle, the main cathodic peak close to 0.1 V is observed during the first cycle, corresponding to the reduction of Mn^2+^ to Mn^0^ and the formation of a solid electrolyte interphase (SEI) layer on the nanocomposite surface^30^. The reduction current peak shifts to 0.6 V since the second cycle, which is ascribed to the formation of Li_2_O and metallic Mn, presented as^22^: MnO + 2 Li → Mn^0^ + Li_2_O. In the oxidation half cycle, the main peak is exhibited at ~1.4 V, in good accord with the oxidation of Mn^0^ to Mn^2+^ and Mn^3+^ in previous reports^9^. Both the reduction and oxidation curves almost overlap with the subsequent ones since the second cycle, indicating excellent electrochemical reversibility of the MnO@C nanocomposite.

Galvanostatic measurements of discharge-charge cycles are further carried out in the MnO@C nanocomposite based on the half-cell configuration at a current density of 0.1 A g^−1^, where several representative cycles, including the 1^st^, 2^nd^, 50^th^, and 100^th^ ones, are displayed (Figure 3b). The voltage drops rapidly to ~0.5 V in the first discharge cycle, followed by two voltage plateaus at 0.5 and 0.3 V. The discharge profile is shifted to 0.6 V since the second cycle. For the charging process, two small voltage plateaus at 1.2 and 2.0 V are observed for all the charging cycles, in good accord with the CV measurement. An ultrahigh capacity of 1542 mAh g^−1^ is recorded for the first discharge process, which decreases to 981 mAh g^−1^ at the first charge process, indicating an initial Coulombic efficiency of 64%. These initial capacities exceed the theoretical value of MnO, which can be ascribed to the decomposition of electrolyte to form the SEI layer and further lithium storage via interfacial charging at metal Li_2_O interface^9^,^22^. In addition, the MnO@C nanocomposite anode presents an excellent cycling performance (Figure 3c, red curve). The discharge capacity becomes much more stable since the second cycle, with the Coulombic efficiency of each cycle over 95%. After 80 cycles, the discharge capacity is well retained at ~800 mAh g^−1^, corresponding to ~82% of that of the second cycle. This result is comparable or better than the best reversible capacity reported previously for MnO_x_-based LIB anodes, such as MnO/C core-shell nanorods^9^ (~600 mAh g^−1^ at 200 mA g^−1^), porous carbon-coated MnO microspheres^22^ (~750 mAh g^−1^ at 50 mA g^−1^), and MnO@C nanocomposite made by copolymerization of poly(acrylonitrile) and Mn oxide precursor containing vinyl groups^26^ (~350 mAh g^−1^ at 0.2C).

To further demonstrate the advantage of direct growth on substrate, MnO@C nanocomposite grown as free-standing power form, but otherwise identical conditions, is coated on Ni foam substrates with binding and conducting additives, and tested as LIB anodes for comparison (Figure 3c, black curve). The galvanostatic measurements at same 0.1 A g^−1^ current density shows an initial discharge capacity of 981 mAh g^−1^, which rapidly drops to 395.6 mAh g^−1^ at the second cycle and is retained at ~177.6 mAh g^−1^ after 80 cycles, corresponding to a capacity retention of 45% compared to that of the second cycle. This comparison clearly indicates that the direct growth of MnO@C nanocomposite over the current collector substantially enhances the Li^+^ storage capacity as LIB anodes. Moreover, the cycling performance of the MnO@C nanocomposite anode is further interrogated, where each step consists of 5 discharge/charge cycles at different current densities in the range of 0.1–2 A g^−1^ (Figure 3d). The discharge capacities are retained at 900, 780, 700, 610 and 520 mAh g^−1^ at the current densities of 0.1, 0.2, 0.5, 1 and 2 A g^−1^, respectively, with a Coulombic efficiency of almost 100% for each cycle. This much enhanced capacity especially at high current rates is contributed to the efficient ion transport through the mesopores in the carbon matrix towards the MnO NP surface, as well as rapid charge transfer to the Ni foam substrate. When the current density is reset to 0.1 A g^−1^, the capacity is recovered to 850 mAh g^−1^, suggesting excellent cycling performance and stability of the MnO@C nanocomposite.

### Supercapacitor

**Figure 4:**
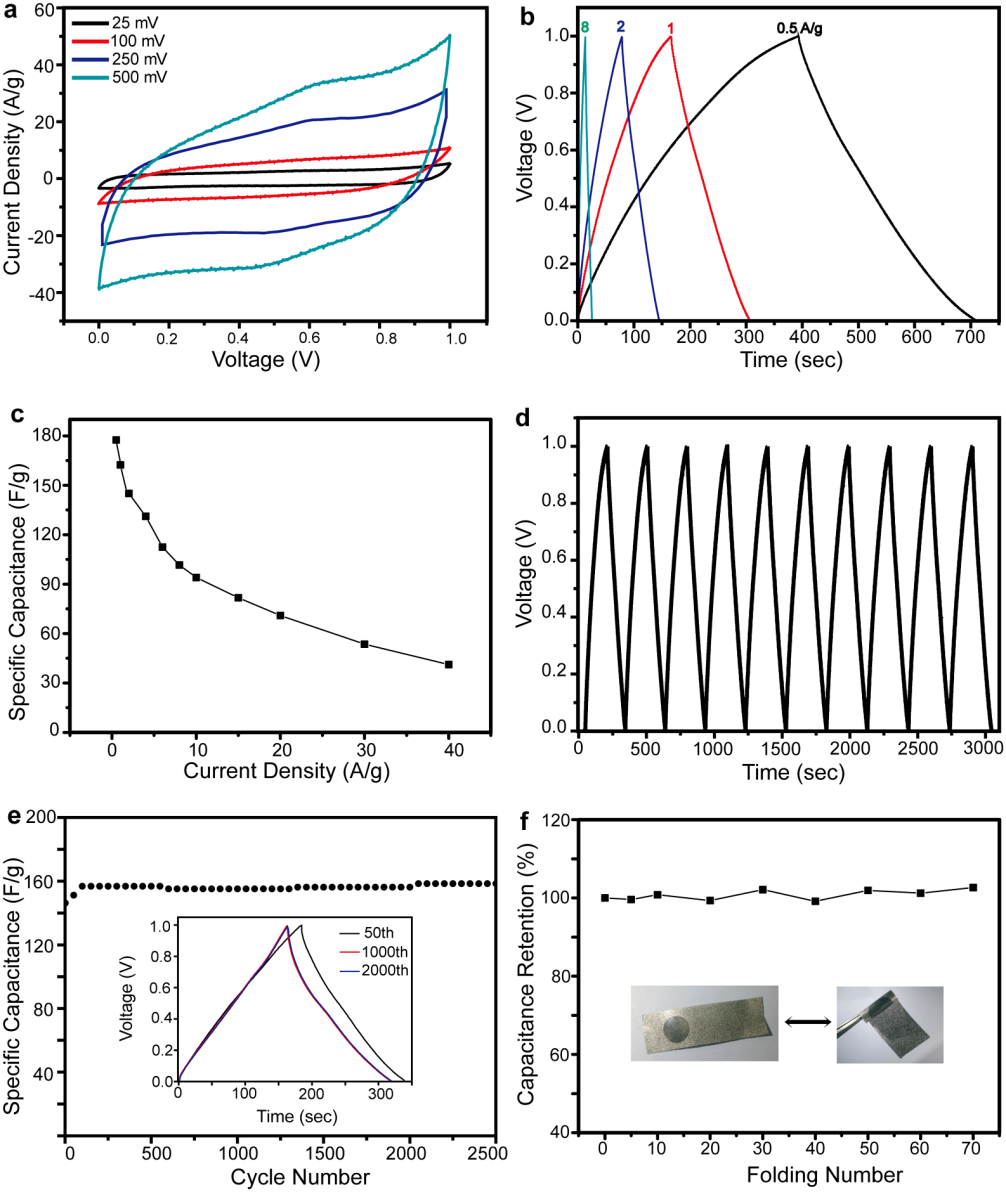
Supercapacitor measurement of MnO@C nanocomposite on Ni foam. (a) Cyclic voltammograms at different scan rate of 25–500 mV s^−1^. (b) Charge-discharge curves at different current densities. (c) Specific capacitance dependence on the current density from 0.5–40 A g^−1^. (d) Repeated charge-discharge curves and (e) capacity retention of 2500 cycles at 1 A g^−1^. Inset: the charge-discharge curves of the 50^th^, 1000^th^ and 2000^th^ cycles. (f) Capacity retention during repeated folding with an angle of almost 180° for 70 times. Inset: optical photos of the folded and extended electrodes of MnO@C nanocomposite on Ni foam.

In addition to LIB anodes, the potential of using the MnO@C nanocomposite directly grown on a conducting substrate as electrochemical capacitors is subsequently evaluated. The MnO@C nanocomposite grown on a Ni foam substrate is fabricated as the working electrode, with a Pt wire serving as the counter electrode. A Na_2_SO_4_ solution is used as the electrolyte, and a voltage range between 0 and 1 V is applied. The CV curves under different scanning rates, including 25, 100, and 250 mV s^−1^, show nearly rectangular feature (Figure 4a), indicating a close-to-ideal pseudocapacitive nature of the electrode. At a high scanning rate of 500 mV s^−1^, the CV curve presents some deviation of a rectangular shape, which can be ascribed to the inherent resistivity of the electrode^31^. The MnO@C nanocomposite exhibits a high specific capacitance of 120 F g^−1^ at a scan rate of 25 mV s^−1^, which decreases to 53 F g^−1^ at a high scan rate of 500 mV s^−1^.

The electrochemical performance of the MnO@C nanocomposite is further evaluated by galvanostatic charge-discharge measurement carried out at different current densities. The charging and discharging curves of several representative current rates, 0.5, 1, 2, and 8 A g^−1^, are exhibited (Figure 4b). All these curves present a symmetrical feature between the charging and discharging branches, suggesting ideal pseudocapacitive nature of fast charge/discharge processes^13^. At 1 A g^−1^, a high specific capacitance of 160 F g^−1^ is obtained, comparable to most of the manganese oxide-based composite materials reported recently, such as MnO_2_/CNT^32^ (179 F g^−1^ at 5 mV s^−1^), MnO_2_/carbon microfiber/CNT^33^ (180 F g^−1^ at 10 mV s^−1^), MnO_2_/graphene oxide nanocomposites^34^ (111 F g^−1^ at 1 A g^−1^), and Mn_3_O_4_ nanorod/graphene^20^ (115 F g^−1^ at 1 A g^−1^). The rate capability is further examined by measuring the charge/discharge cycles at higher current densities (Figure 4c). The specific capacitance shows a decrease trend with the increase of current density, due to the diffusion-limited charge/discharge process as well as the electrode overpotential at high current densities^35^, while it still maintains good capacitance retention. The specific capacitances at 8 and 40 A g^−1^ are 101.6 and 41.2 F g^−1^, corresponding to 63.5% and 25.8% of the value obtained at 1 A g^−1^, suggesting attractive rate capabilities for potential high power applications. Our result is comparable or better than most of the manganese oxide-based composite materials reported in similar high current densities, such as MnO_2_ coaxially coated on aligned carbon nanofiber arrays^36^ (70 F g^−1^ at 15 A g^−1^), and Mn_3_O_4_ nanorod/graphene sheet composites^20^ (88 F g^−1^ at 10 A g^−1^). Another recent report of MnO_2_ NW/mesoporous carbon/MnO_2_ NPs^21^ presents a high specific capacitance of 150 F g^−1^ at 60 A g^−1^, while this approach requires separate growth and coating steps for each structural component. In comparison, our synthesis approach has only a single coating and calcination step, which is much more convenient and readily to scale up.

In addition to high specific capacitance, the cycle stability is further tested to demonstrate its potential for long-term use. The charge-discharge cycles of the MnO@C nanocomposite at a current density of 1 A g^−1^ exhibits repeated, almost identical triangular curve shapes (Figure 4d). The long-term stability is demonstrated by the specific capacitance as a functional of cycle numbers (Figure 4e). After 2500 cycles, the specific capacitance is retained at ~160 F g^−1^, corresponding to ~110% of its original value. The slight increase of capacitance is ascribed to the activation effect of electrochemical cycling, suggested by previous reports of other manganese oxide-based electrode materials^14^. This cycling performance is better than previous reports of MnO_2_-based composites, such as graphene oxide-MnO_2_ nanocrystals^34^, which show over 84% capacity retention after 1000 cycles. A main reason of the capacitance loss for manganese oxide-based supercapacitor is the dissolution of active materials into electrolyte solution during cycling^37^. However, in our experiment, the electrolyte remains transparent after the cycling test, indicating that the majority of the MnO is stable and not dissolved. Moreover, the mechanical stability of the MnO@C nanocomposite on Ni foam is demonstrated by measuring of electrochemical performance after repeated folding (Figure 4f). The specific capacitance is retained almost constant (>96%), even after being folded with an angle of almost 180° for 70 times. These results suggest that the direct growth of MnO@C nanocomposite on substrates present remarkable electrochemical and mechanical stability.

### Sensor

The MnO@C nanocomposite, due to its open mesopores for fast transport of molecules and enhanced electron transfer through the carbon matrix towards substrates, offers not only high electrochemical energy storage capacities, but also can serve as a sensitive platform for detection of chemical or biological species that indicate specific cellular process^38^. H_2_O_2_ is one of the most important small molecule targets that are related to many cell functions^25^, and has been recently reported as a potential marker for tumor cells^39^. However, the direct measurement of H_2_O_2_ from cellular process by manganese oxide-based sensors has not been demonstrated.

**Figure 5:**
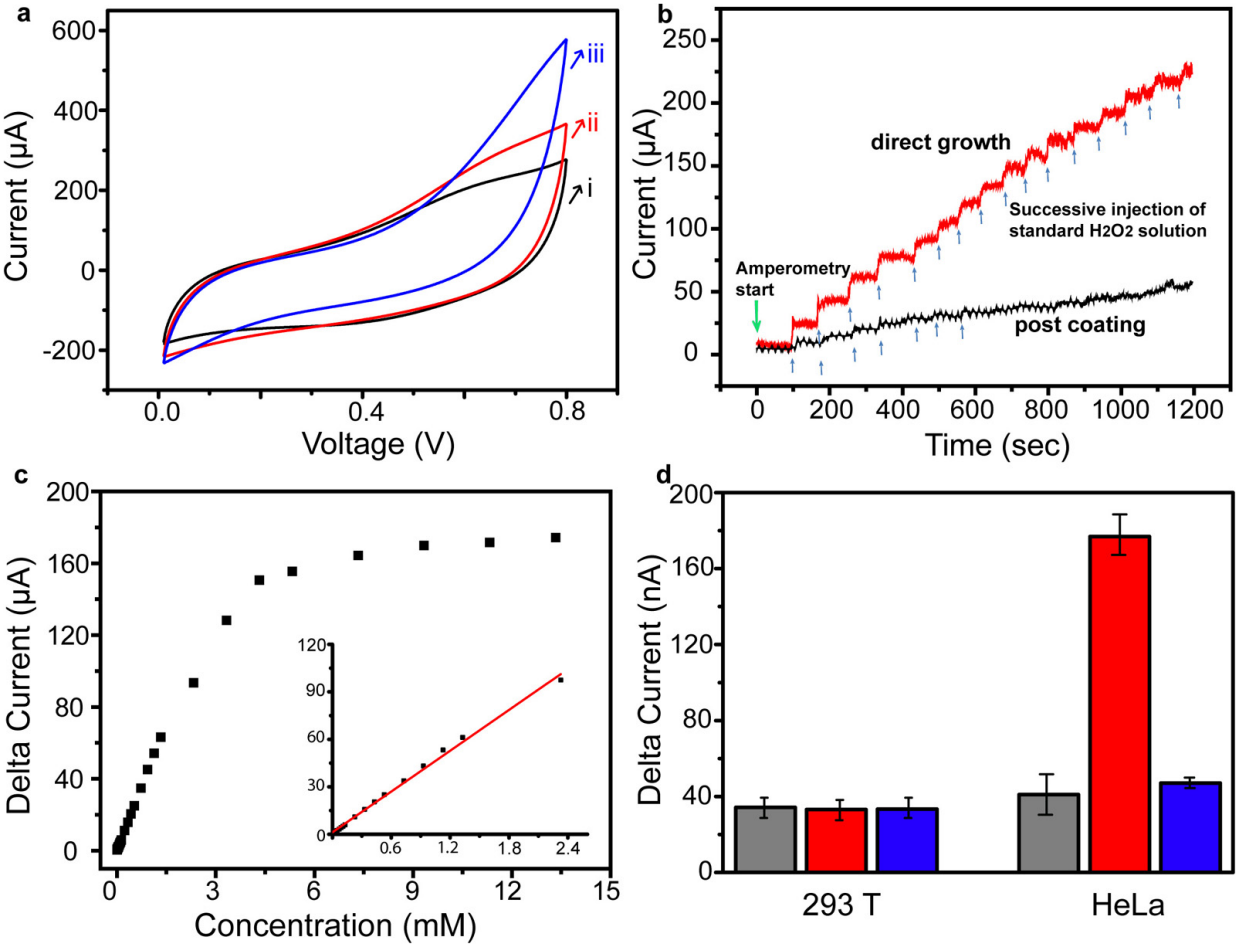
Sensing and cellular measurement of MnO@C nanocomposite on Ti foil. (a) Cyclic voltammograms in (i) 0, (ii) 0.4, and (iii) 2 mM of H_2_O_2_ in PBS solution. (b) Current-versus-time plot with repeated addition of 200 μM of H_2_O_2_ for directly growth (red curve) and post-coating (black curve) of MnO@C nanocomposite on Ti foil. (c) Concentration dependence plot of current change at different H_2_O_2_ concentrations. Inset: linear fitting for concentration range of 2 μM–2.4 mM. (d) Cellular assay of H_2_O_2_ detection for 293T cells and HeLa cells. Three conditions are presented for each cell lines: buffer without PMA or catalase (grey bars), buffer with PMA only (red bars), and buffer with both PMA and catalase (blue bars).

In our experiment, the CV of the MnO@C nanocomposite grown on a Ti substrate is first measured, in the presence of 0.4 and 2 mM of H_2_O_2_ in a phosphate buffer solution, respectively (Figure 5a). Compared with the CV curve measured without H_2_O_2_, a substantial increase of the current density is observed, indicating the increase charge transfer upon the addition of H_2_O_2_. In order to optimize the signal-to-noise ratio of the subsequent time-dependent current measurement, the bias range is selected as 0.6–0.7 V, where the current baseline of MnO@C nanocomposite without H_2_O_2_ is close to zero and the current increase with the H_2_O_2_ addition is relatively large (Figure S5). The response of the MnO@C nanocomposite to H_2_O_2_ is then interrogated by the time-dependent current measurement, with successive injection of H_2_O_2_ at intervals under a bias of 0.65 V (Figure 5b). Upon each addition of 200 μM of H_2_O_2_, the MnO@C nanocomposite electrode responds quickly with a conductance increase, which reaches equilibrium within 5–10 s. The magnitude of current increase for the subsequent H_2_O_2_ injections is smaller than that for the first several H_2_O_2_ injections, suggesting the signal saturation at higher H_2_O_2_ concentrations. Interestingly, when the free-standing MnO@C nanocomposite is coated on a Ti substrate, a much less response to the same H_2_O_2_ injection is recorded, which also shows earlier saturation upon the successive addition of H_2_O_2_, suggesting the importance of direct growth/attachment of MnO@C nanocomposite on the conducting substrate. The conductance change with different H_2_O_2_ concentrations (2, 10, 20, 100, 200 and 1000 μM) and the corresponding calibration curves are exhibited in Figure S6 and 5c, respectively. A wide linear range of 2 μM–2.4 mM is obtained, with the lowest H_2_O_2_ concentration detected as ~2 μM. These values are comparable or superior to most of the enzymatic or non-enzymatic manganese oxide-based H_2_O_2_ sensors^25^.

The MnO@C nanocomposite is further used for electrochemical detection of H_2_O_2_ produced by living cells, including human embryonic kidney (HEK) 293T cells (a normal cell line) and HeLa cells. A low concentration (1 μg ml^−1^) of phobol 12-myristate-13-acetate (PMA) is added to the cell culture for a short period of time (30–60 s), which can induce H_2_O_2_ generation from tumor cells^40^, and then a small amount of the cell culture solution containing H_2_O_2_ is added to the electrochemical detection assay (Methods). For the 293T cells (~10^5^ cells/mL), the MnO@C nanocomposite electrode does not show an observable amperometric response before and after the addition of PMA. Under otherwise identical conditions, a substantial larger signal is observed from the MnO@C nanocomposite electrode for HeLa cells (~10^5^ cells/mL) incubated with PMA. Furthermore, the introduction of a catalase into the HeLa cell culture medium reduces the current change to the background level. As catalase is known to selectively decompose H_2_O_2_^39^, this result indicates that the current increase of the MnO@C nanocomposite electrode is attributed to the formation of H_2_O_2_ by the cellular process. Moreover, the higher signal from HeLa cells suggests a more active cellular activity than that of the 293T normal cells, in good accord with previous reports^39^,^41^. These results suggest the potential use of the highly sensitive MnO@C nanocomposite electrode for detection of cellular functions.

## Discussion

The direct growth method for the MnO@C nanocomposite provides a facile and efficient means of synthesizing mono-dispersed, ~10-nm-diameter MnO NPs embedded in mesoporous carbon coating, which is directly attached to the conducting substrate (current collector) for efficient charge transport. In addition, the loading amount can be conveniently controlled by repeated coating of the Mn-oleate precursor solution on the substrate, followed by a single calcination step to convert to the MnO@C nanocomposite. The excellent performances of the MnO@C nanocomposite as LIB anodes, supercapacitors, and chemical sensors are attributed to the following advantages. First, the mesopores in the carbon matrix facilitates fast transport of molecules and ions from the electrolyte solution to the MnO NP surface. Second, the monodispersed, ultra-small MnO NPs and the surrounding mesoporous carbon matrix provide a high surface area for electrochemical reactions, which can sufficiently utilize the active materials. Third, the carbon matrix offers an efficient charge transport pathway from the MnO NPs to the supporting substrate, where the direct growth of the MnO@C nanocomposite on the conducting substrate surface allows for low contact resistance and enhanced charge transfer. Finally, the carbon matrix coating prevents the MnO NPs from degradation, while at the same time, the mesopores also serve as structural buffers for the dramatic volume change during Li^+^ intercalation/extraction or mechanical deformation.

**Figure 6:**
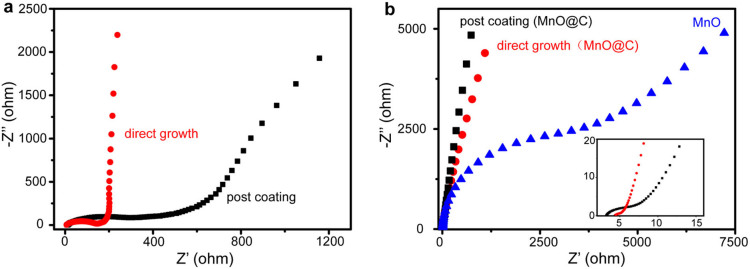
(a) Nyquist plots of MnO@C nanocomposites as LIB anodes, for the direct growth method (red curve) and the post-coating method (black curve). (b) Nyquist plots of MnO@C nanocomposites as supercapacitor anodes, for the direct growth method (red curve), the post-coating method (black curve), and after removal of carbon coating for the direct grown thin films (blue curve). Inset: close up of the high frequency region.

To confirm the enhanced charge transport by the carbon coating, the electrochemical impedance spectroscopy (EIS) is carried out for the MnO@C nanocomposite directly grown on Ni foam and fabricated as LIB anodes (Figure 6a), compared to that of the coating of pre-synthesized MnO@C nanocomposite on identical Ni foam substrates. The Nyquist plots are recorded at a frequency range of 0.01 Hz–100 kHz at an amplitude of 10 mV. The MnO@C nanocomposite grown on Ni foam exhibits a much smaller diameter of the depressed semicircle, indicating a much more efficient charge transfer process at the electrode interface^7^,^42^,^43^. Based on an equivalent electrical circuit model for LIB^44^, the charge transfer resistance for the direct growth and the post-coating approach are ~123.3 and ~204.8 Ω, respectively. Similarly, for the MnO@C nanocomposite fabricated as supercapacitor electrodes, the direct growth method provides a much smaller diameter of the Nyquist plot than that of the conventional post coating method (Figure 6b). The equivalent circuit modeling^45^ yields the charge transfer resistances of ~1.2 and ~1.8 Ω, respectively. Moreover, when the MnO@C nanocomposite is calcined to remove the carbon matrix, the Nyquist plot shows a substantially increased diameter of the semicircle, corresponding to a charge transfer resistance of 709.5 Ω. This result suggests that the removal of the carbon coating leads to a much increased electrical impedance and a reduced charge transfer process.

In summary, we have demonstrated a facile, two-step coating/calcination method to synthesize a MnO@C nanocomposite, by directly coating of the Mn-oleate precursor solution on arbitrary conducting substrates, such as Ni foam and Ti foil, followed by calcination to convert the organic precursor molecules into a matrix of MnO NPs and mesoporous carbon. The monodispersed, sub-10 nm-diameter MnO NPs serve as the main sites of the electrochemical reaction, and the carbon matrix provides an efficient charge transport pathway from MnO NPs to the underlying substrate. In addition, the mesopores inside the carbon matrix also lead to fast mass transport of molecules and ions towards the MnO NP surface, as well as the structural spacer for the volume change during the lithiation/delithiation or mechanical deformation. This MnO@C nanocomposite has exhibited excellent performance for electrochemical energy storage and sensing. The LIB anodes made of the MnO@C nanocomposite on Ni foam show a high reversible capacity of ~800 and ~520 mAh g^−1^, at 0.1 and 2 A g^−1^, respectively. The supercapacitor electrodes made of the MnO@C nanocomposite on Ni foam present an electrochemical capacitance of 160 and 41 F g^−1^, at 1 and 40 A g^−1^, respectively. The electrochemical sensors based on the MnO@C nanocomposite on Ti foil show a wide, linear response regime for H_2_O_2_, with detection limit as low as 2 μM. In addition, H_2_O_2_ produced by HeLa cells can be well detected, clearly distinguished from that obtained from normal cell lines. Moreover, this synthesis approach is facile and convenient, and can be applied for other transition metal oxide NP@mesoporous C nanocomposite on a variety of substrates, thus opening up substantial opportunities for many promising electrochemical applications.

## Methods

### Synthesis of MnO@C nanocomposites

The Mn-oleate precursor was prepared by a simple chemical reaction of MnCl_2_ and sodium oleate, modified from a previous report^27^. In brief, 0.80 g of MnCl_2_·6H_2_O (~2 mmol) and 2.44 g of sodium oleate (~4 mmol) were first dissolved in a mixture of H_2_O (6 mL), ethanol (8 mL) and hexane (14 mL), with continuous stirring at room temperature for 30 min. The color of the upper solution gradually changed to light brown. The resultant mixture was kept still and aged at 70°C in an oven for 4 h. Afterwards, the upper solution (organic phase) was collected, and washed with deionized (DI) water for several times to obtain the Mn-oleate/hexane solution. To prepare for the MnO@C nanocomposite, a conductive substrate (such as Ni foam or Ti foil) was dipped into the Mn-oleate/hexane solution for several seconds. After the solvent was evaporated at room temperature, the substrate was coated with a red-brown waxy solid. This step can be repeated for several times to increase the loading amount of the Mn-oleate precursor. The substrate was then heated to 550°C at 10°C min^−1^ under Ar atmosphere, and then kept for 2 h before cooling to room temperature.

The growth mechanism was proposed elsewhere^27^. In brief, the metal oleate, such as Mn(oleate)_2_, is formed first in the solution reaction, in which Mn is +2. The oleate ligands are thermally dissociated into CO_2_, thus remaining in MnO. In addition, the mesopores are formed during the formation/elimination of CO_2_ from the reactants.

The MnO percentage in the nanocomposite was determined by ICP, briefly described as follows: an as-made MnO@C nanocomposite sample was mixed with H_2_SO_4_ (1 M) to fully dissolve the MnO content, and the supernatant was collected and measured by ICP, which showed the concentration of Mn^2+^. In addition, no other metal ions were detected by ICP, suggesting the purity of our samples. The mass percentage of MnO was then calculated based on the measured Mn^2+^ concentration and the original sample mass.

### Cell culture

Cells were cultured in 10 ml of DMEM (high glucose) supplemented with 10% fetal bovine serum and 1% penicillin/streptomycin GIBCOBRL (Grand Island, New York, USA), at 37°C in a humidified hood filled with 5% CO_2_. After reaching 80–90% confluence, the cells were adjusted in PBS for 2 h prior to the sensing experiment. For the cell number counting, the cells were lifted with trypsin-EDTA and then re-dispersed in DMEM (high glucose) medium. The cell number was counted using a hemocytometer.

### Electrochemical measurement

The mass loadings of the actual samples for lithium-ion battery and supercapacitor tests were in the range of 1.6 ± 0.2 mg/cm^2^. For lithium-ion battery measurement, the MnO/C nanocomposite electrodes were galvanostatically cycled on a galvanostat over a voltage range of 3.0–0.01 V vs. Li^+^/Li. Cyclic voltamograms (CVs) were recorded on a potentiostat over a voltage range of 3.0–0.01 V vs. Li^+^/Li at a scan rate of 0.5 mV/s. In rate capability test, the lithiation and delithiation current densities were changed every five cycles, according to this sequence of values: 100, 200, 500, 1000, 2000, and 100 mA g^−1^.

For supercapacitor measurement, the electrochemical measurements were conducted using a three-electrode mode in a 0.5 M Na_2_SO_4_ solution. The working electrodes were prepared by directly grow on MnO@C nanocomposites on a Ni foam (1.2 cm in diameter), followed by pressing the substrate onto another Ni foam with larger size (2 cm × 5 cm). The reference electrode and counter electrode were Ag/AgCl electrode and Pt wire, respectively. Typical CV curves were measured between 0.01 and 1 V.

For the H_2_O_2_ sensing, the MnO/C nanocomposite electrodes were galvanostatically cycled on a galvanostat over a voltage range of 3.0–0.01 V vs. an Ag/AgCl reference electrode. The time-dependent conductivity test was carried out in a 5 mL of PBS solution at a bias voltage of 0.6–0.7 V, with addition of different concentrations of H_2_O_2_ in PBS. For the cellular measurement, both HEK 293T cells and HeLa cells were incubated with 1 μg mL^−1^ PMA (Sigma-Aldrich, USA) for 30 s. For the catalase inhibition, 1 mL of a catalase solution (350 unit mL^−1^) was added into the cell culture for 30 min, before the addition of PMA. The measurement of the MnO@C nanocomposite was carried out in the same solution as the cell culture medium. Then 100 μL of the cell culture was injected into the detection solution (5 mL) for the conductivity measurement.

## Author Contributions

T.W. carried out all the experiments and wrote the paper. Z.P. helped in the supercapacitor measurement. Y.W. helped in the lithium-ion battery measurement. J.T. helped in the cell culture and sensing measurement. G.Z. supervised the research and revised the manuscript.

## Supplementary Material

Supplementary Information Supplementary information

## Figures and Tables

**Figure 1:**
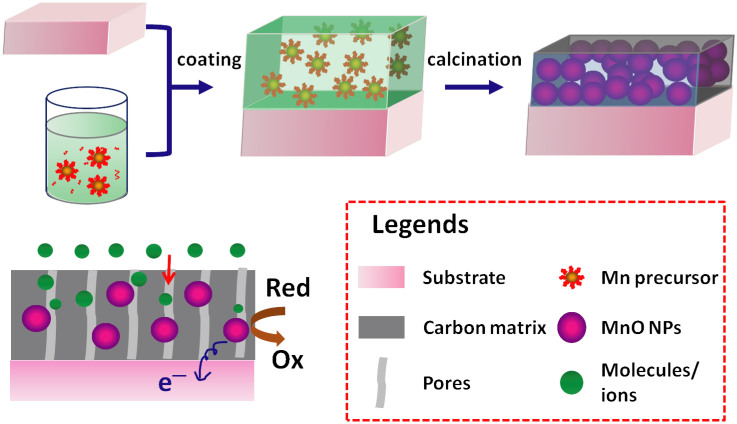
Schematic of preparation of Mn-oleate precursor, direct coating of precursor solution on a conducting substrate, and thermal calcination to directly grow MnO@C nanocomposite on the substrate surface.
