# Supplementary material for: MnO Nanoparticle@Mesoporous Carbon Composites Grown on Conducting Substrates Featuring High-performance Lithium-ion Battery, Supercapacitor and Sensor
Source: Sci Rep. 2013 Sep 18;3:2693. doi: 10.1038/srep02693 (PMC3776197; doi:10.1038/srep02693)
Supplement: Supplementary Information [file srep02693-s1.doc]

**Supporting Information**

MnO Nanoparticle@Mesoporous Carbon Composites Grown on Conducting Substrates Featuring High-performance Lithium-ion Battery, Supercapacitor and Sensor

Tianyu Wang^1^, Zheng Peng^1^, Yuhang Wang^1^, Jing Tang^1^, and Gengfeng Zheng^1^,*

^1^ Laboratory of Advanced Materials, Department of Chemistry, Fudan University, Shanghai, 200433, People’s Republic of China

* Corresponding author: gfzheng@fudan.edu.cn (G. Z.)

This file contains:

Supporting Figures S1—S6


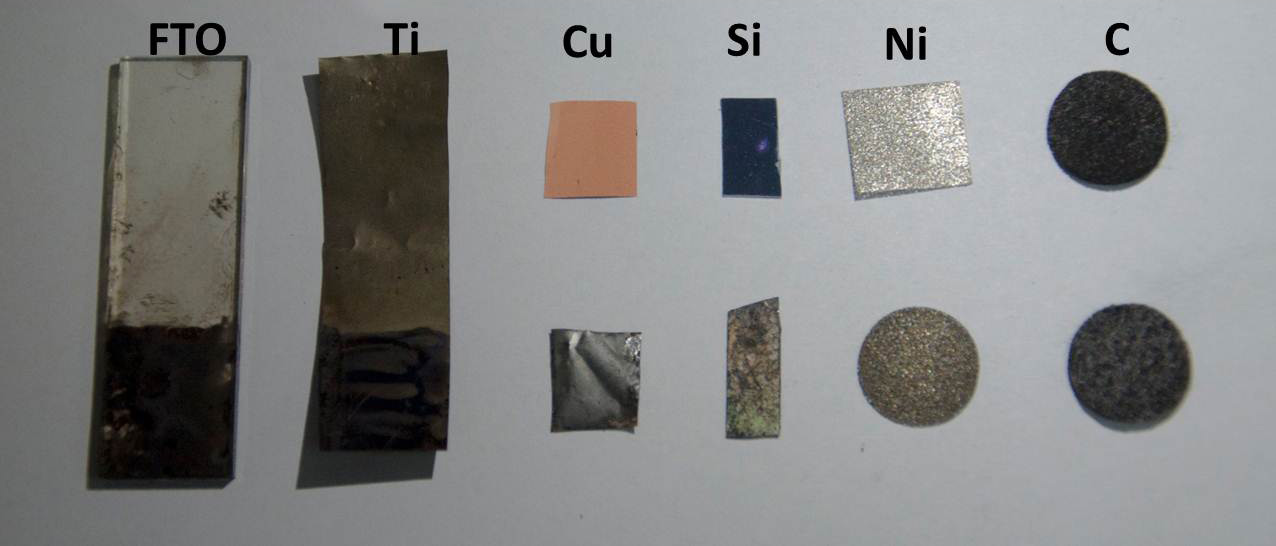


Figure S1. Digital photos of the MnO@C nanocomposites coated on different substrates. Top: substrates before coating; Bottom: substrates after coating and calcination.


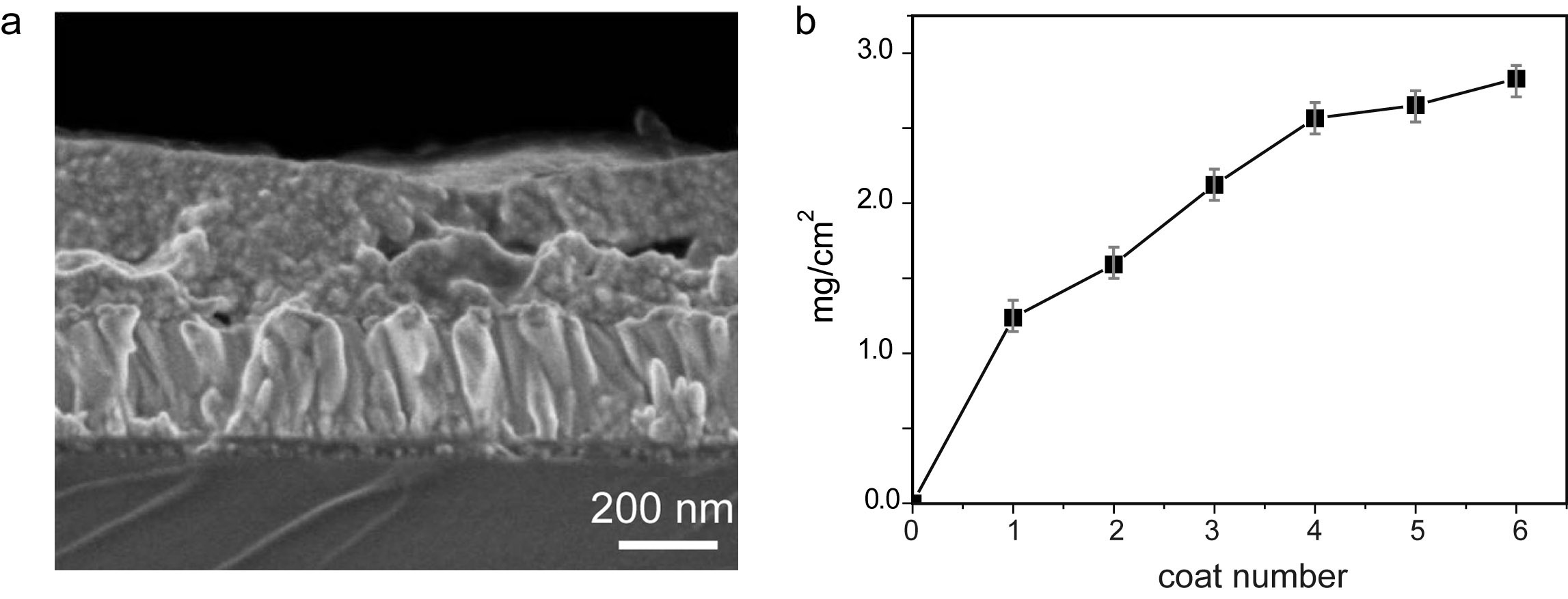


Figure S2. (a) Cross-section SEM image of a MnO@C nanocomposite thin film on substrate. (b) Mass per area of MnO@C nanocomposite directly grown on Ni substrates vs. coating times.


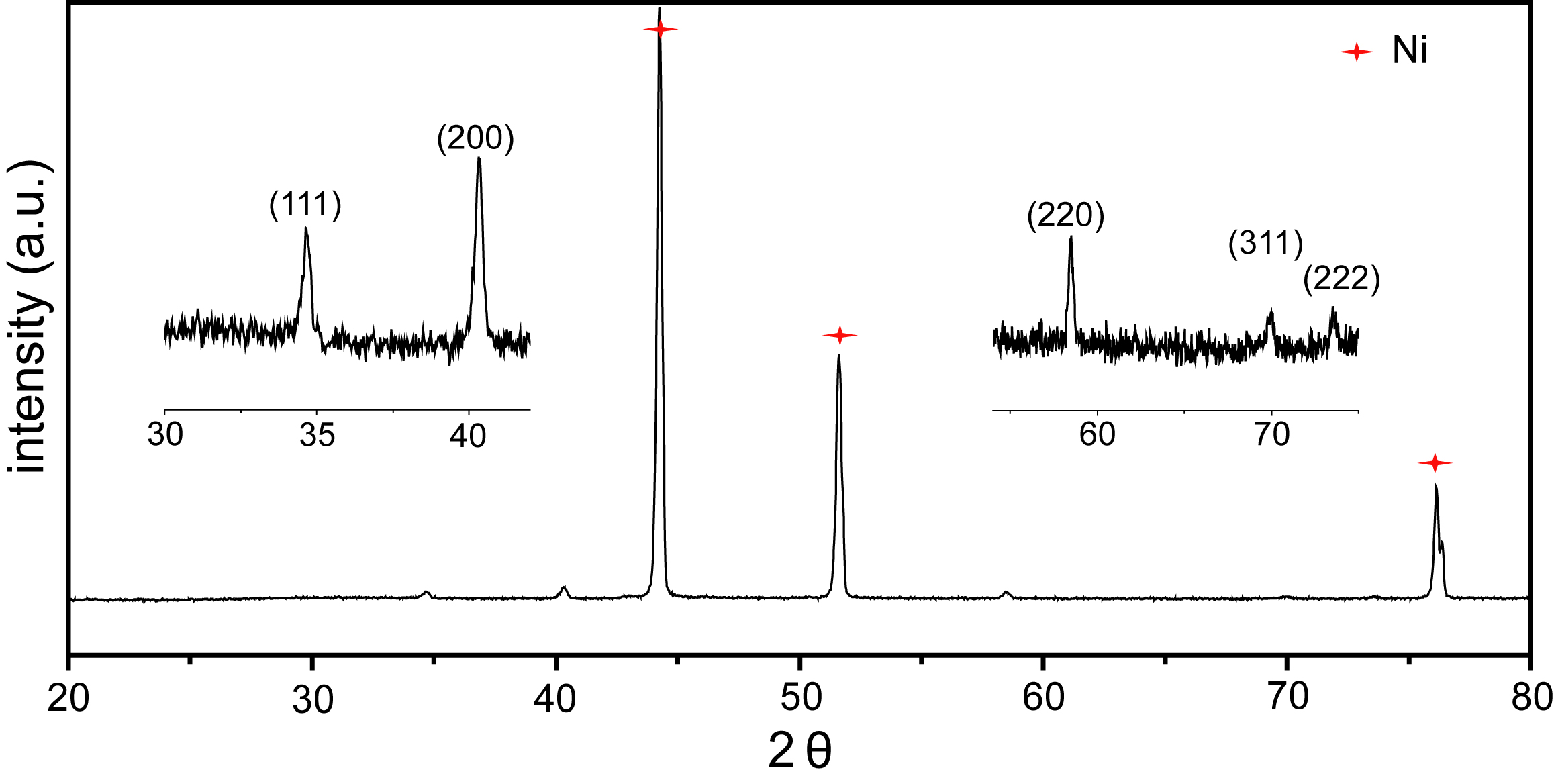


Figure S3. Full XRD pattern (same as Fig. 2e) of MnO@C nanocomposite on a Ni foam substrate. The diffraction peaks of the Ni foam substrate are marked with asterisks.


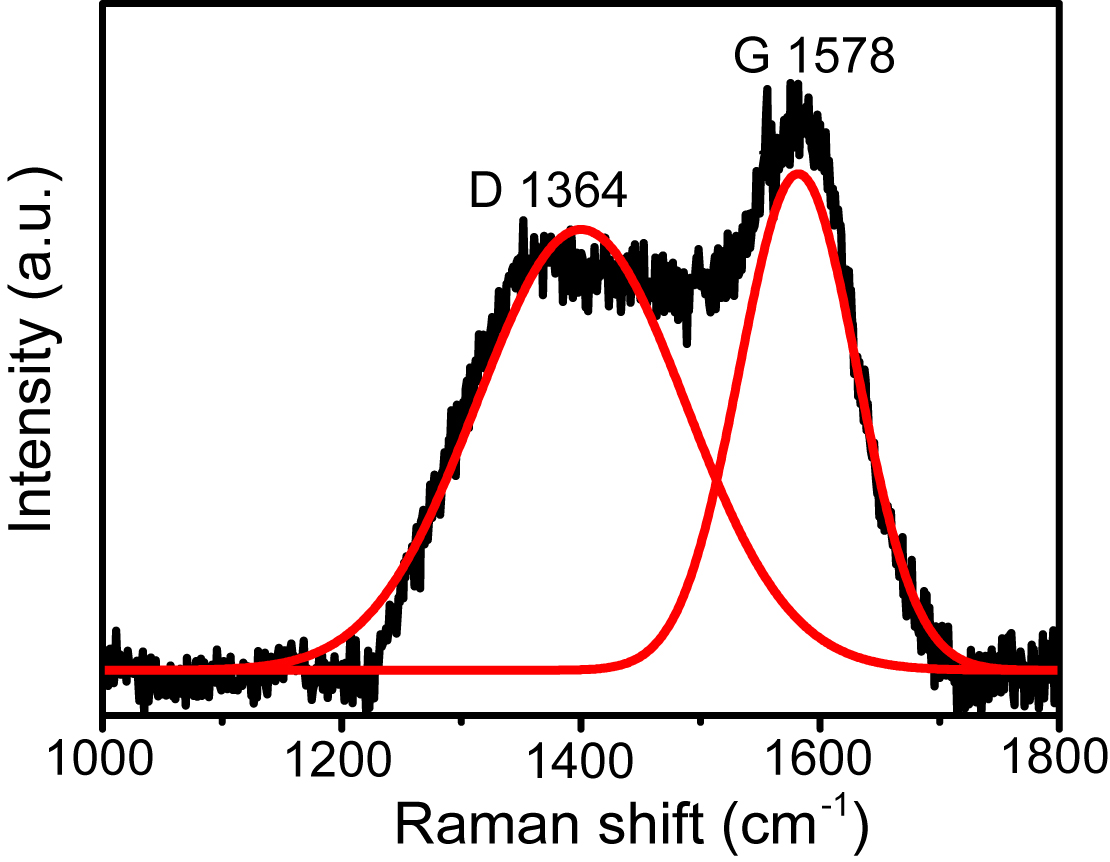


Figure S4. Raman spectra between 1000 and 1800 cm^-1^ showing the D and G bands for carbon.


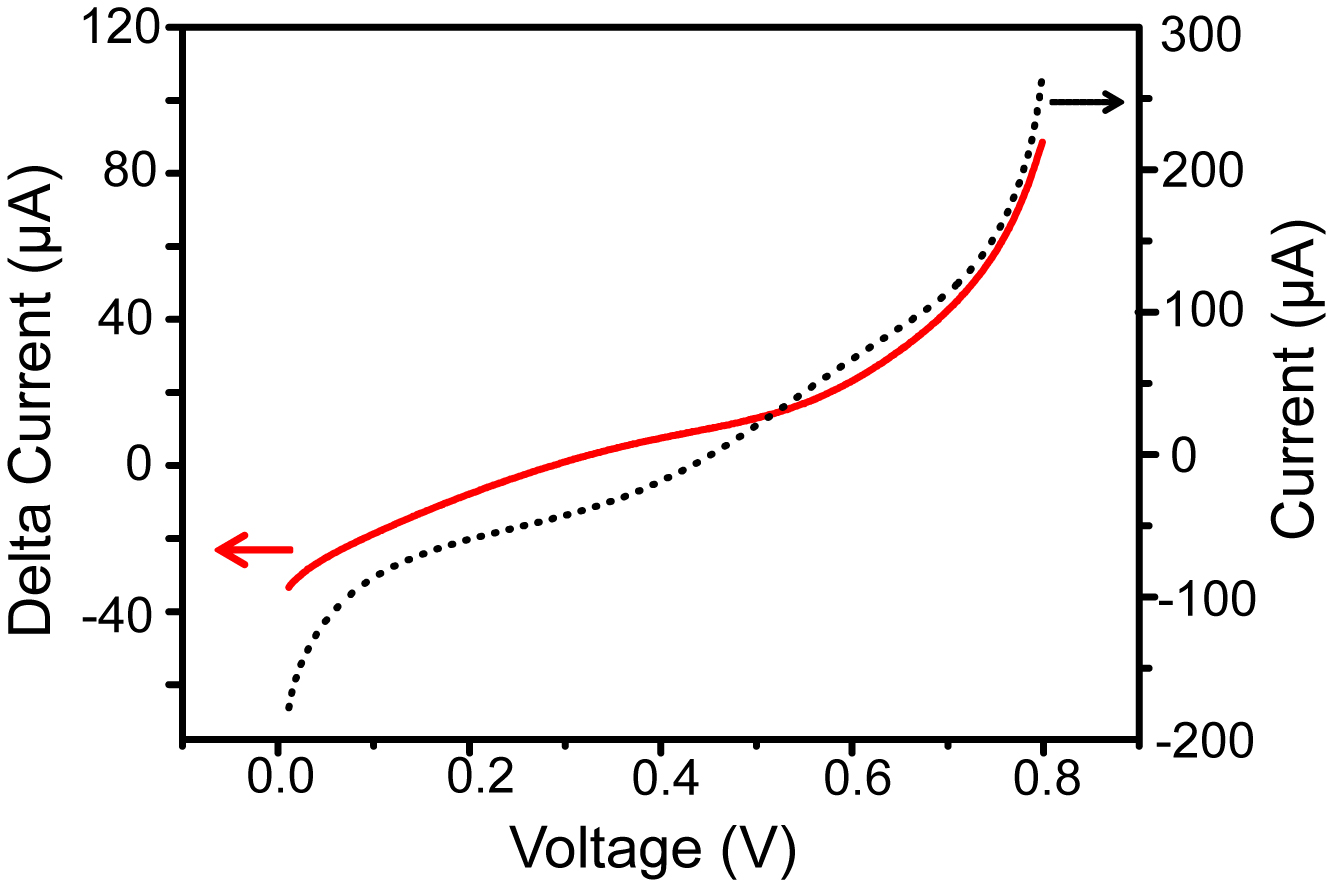


Figure S5. Black dot curve: average cathodic and anodic current of MnO@C nanocomposite in PBS without H_2_O_2_. Red curve: Difference in current (∆Current) between 0 and 0.4 mM H_2_O_2_.


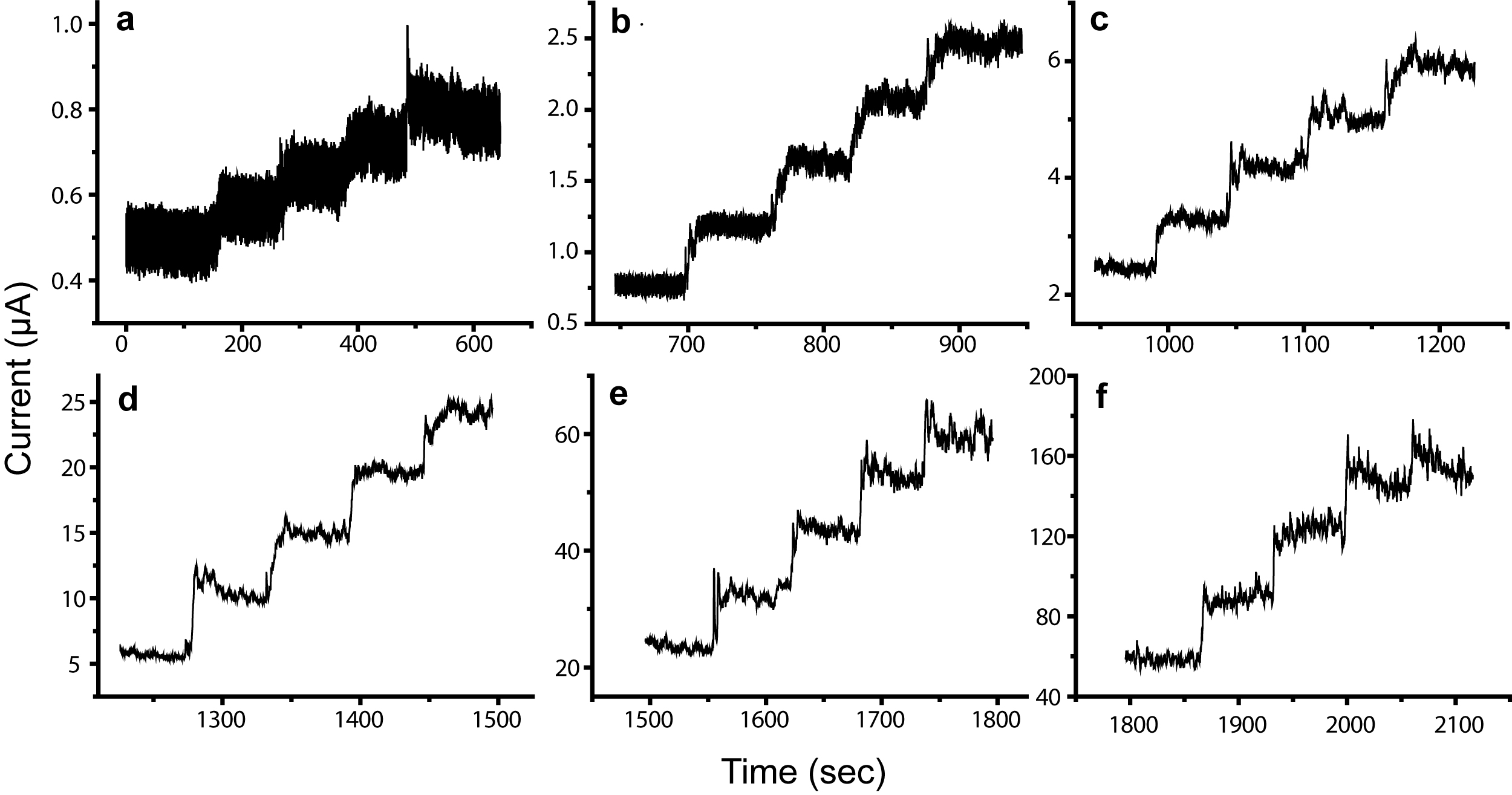


Figure S6. MnO@C nanocomposite directly grown on Ti foil for detection of different H_2_O_2_ concentrations: (a) 2 µM, (b) 10 µM, (c) 20 µM, (d) 100 µM, (e) 200 µM, (f) 1000 µM of H_2_O_2_.
